# Combined Effect of Ceramic Waste Powder Additives and PVA on the Structure and Properties of Geopolymer Concrete Used for Finishing Facades of Buildings

**DOI:** 10.3390/ma16083259

**Published:** 2023-04-20

**Authors:** Evgenii M. Shcherban’, Alexey N. Beskopylny, Sergey A. Stel’makh, Levon R. Mailyan, Besarion Meskhi, Alexandr A. Shilov, Elena Pimenova, Diana El’shaeva

**Affiliations:** 1 Department of Engineering Geology, Bases, and Foundations, Don State Technical University, 344003 Rostov-on-Don, Russia; au-geen@mail.ru; 2 Department of Transport Systems, Faculty of Roads and Transport Systems, Don State Technical University, 344003 Rostov-on-Don, Russia; 3 Department of Unique Buildings and Constructions Engineering, Don State Technical University, Gagarin Sq. 1, 344003 Rostov-on-Don, Russia; sergej.stelmax@mail.ru (S.A.S.); lrm@aaanet.ru (L.R.M.); alexandr_shilov@inbox.ru (A.A.S.); diana.elshaeva@yandex.ru (D.E.); 4 Department of Life Safety and Environmental Protection, Faculty of Life Safety and Environmental Engineering, Don State Technical University, 344003 Rostov-on-Don, Russia; spu-02@donstu.ru; 5 Department of Architecture, School of Architecture, Design and Arts, Don State Technical University, 344003 Rostov-on-Don, Russia; spu-57.1@donstu.ru

**Keywords:** geopolymer concrete, sustainable concrete, green materials, building’s facade, ceramic waste powder

## Abstract

Currently, there is great interest in geopolymer composites as an alternative and environmentally friendly basis for compositions for restoring the facades of historical and modern buildings. Although the use of these compounds is much smaller than conventional concrete, replacing their main components with ecological geopolymer counterparts still has the potential to significantly reduce the carbon footprint and reduce the amount of greenhouse gas emitted into the atmosphere. The study aimed to obtain geopolymer concrete with improved physical, mechanical, and adhesive characteristics, designed to restore the finishing of building facades. Regulatory methods, chemical analysis, and scanning electron microscopy were applied. The most optimal dosages of additives of ceramic waste powder (PCW) and polyvinyl acetate (PVA) have been established, at which geopolymer concretes have the best characteristics: 20% PCW introduced into the geopolymer instead of a part of metakaolin, and 6% PVA. The combined use of PCW and PVA additives in optimal dosages provides the maximum increase in strength and physical characteristics. Compressive strength increased by up to 18%, bending strength increased by up to 17%, water absorption of geopolymer concretes decreased by up to 54%, and adhesion increased by up to 9%. The adhesion of the modified geopolymer composite is slightly better with a concrete base than with a ceramic one (up to 5%). Geopolymer concretes modified with PCW and PVA additives have a denser structure with fewer pores and microcracks. The developed compositions are applicable for the restoration of facades of buildings and structures.

## 1. Introduction

Despite the growth in the pace of current construction, an integral part of the construction industry is the maintenance and restoration of already-built buildings [1]. As a rule, subject to building codes and regulations during the construction of a building or structure, a building’s main load-bearing structures work reliably for the entire period of operation. Problems with them can arise only when the loading conditions, the design scheme, and the increase in the level of operational loads change. This can happen due to redevelopment or the rearrangement of large equipment.

Additionally, damage to load-bearing structures can occur due to emergencies, earthquakes, fires, etc. In the absence of these factors, the main load-bearing structures of the building do not require repair and restoration; however, facade elements are daily exposed to atmospheric and other influences that accelerate their wear. This is especially true for historical buildings [2,3]. In most of these buildings, first, brickwork and ceramic elements of the facade are subjected to heavy wear.

Repair of damaged brickwork is most often carried out using cement-based mortars [4]. However, the use of Portland cement for large-scale use in the field of repair of elements of building facades does not correspond to the concept of sustainable development, since the production of cement is associated with high energy costs and a large amount of greenhouse gas emissions [5,6]. This makes it relevant to search for affordable and environmentally cleaner alternatives to compositions for the repair of brickwork and building facade elements based on Portland cement with the same or better physical and mechanical characteristics [7].

The use of geopolymers to produce such compositions is a more environmentally friendly practice than is the case with conventional Portland cement [8]. The use of geopolymers is much less energy-intensive than cement production due to the use of industrial byproducts and leaves a much smaller carbon footprint than the extraction and production of concrete mix components based on conventional Portland cement [9,10,11]. Additionally, geopolymer concretes are not inferior to classical compositions for repairing brickwork and facade elements in terms of strength and performance characteristics [2,12,13,14]. Geopolymer concretes have higher off-axial and flexural tensile strengths, as well as outstanding resistance to high temperatures and aggressive environmental factors [15,16]. The above makes geopolymer concretes a prime candidate as an alternative to Portland cement-based compositions traditionally used for repairing brickwork and facade elements [17].

Despite the above advantages, geopolymers have not yet gained great popularity as materials for the repair and restoration of brickwork and elements of building facades, leaving the advantage in this niche to mortars based on ordinary Portland cement [18,19,20]. The main reason for this is the small amount of practical experience in the use of such compositions for this purpose. Geopolymer concretes used for the repair and restoration of brickwork and facade elements should have reduced brittleness and increased adhesion to restored stone and ceramic materials [21,22]. An important role is occupied by the issue of chemical and structural interaction of the composite with the restored surface [23,24]. 

**Figure 1:**
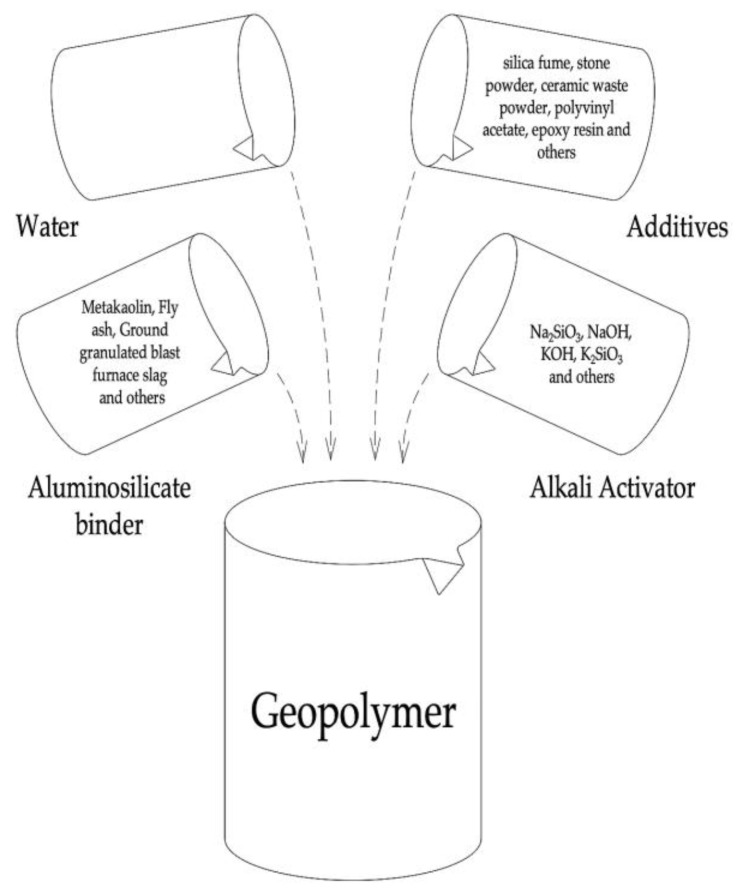
The main components of the geopolymer composition.

It should also be added that in matters of repair and restoration of elements of facades of buildings, until now, the research problems were the physical and mechanical characteristics of the compositions and their aesthetic properties. Today, this traditional approach, ignoring energy savings and reducing the carbon footprint, has been replaced by an opposite one, which is based on the principles of eco-design, which involve the integration of environmental aspects into all stages of the design process [25,26]. Due to these factors, today there is a growing interest of researchers in geopolymer compositions as environmentally friendly and sustainable materials for use in the field of construction and architecture [27,28,29]. The scheme of the main components of the geopolymer composition is shown in Figure 1.

Aluminosilicate binders, which are the basis of geopolymer and alkali-activated compositions, are the main components of the earth’s crust [30,31,32,33,34,35]. These raw materials provide competitive properties of geopolymer concrete based on them, such as thermal stability, low shrinkage, frost resistance, resistance to an open flame and aggressive environments, durability, and recyclability [36,37,38,39]. In addition, industrial waste, such as fly ash, rice husk, granulated metallurgical slag, and so on, can often act as aluminosilicate raw materials for geopolymer concretes, which creates favorable conditions for integrating the process of mass and environmentally friendly disposal of industrial waste into the process of manufacturing geopolymer concretes [32,40,41,42,43,44]. The effective use of construction waste in the form of ceramics [9,28,45], in particular recycled bricks [39], in geopolymer compositions for the restoration of building facades is known. Such compositions have improved adhesion properties to various facing materials, such as concrete and ceramics [45,46], and effectively fill cracks and voids, as well as glue surfaces. In the framework of the study [45], the authors proposed a method to produce new mortars based on waste from the production and culling of ceramics. The resulting compositions showed a more uniform microstructure with fewer microcracks than conventional geopolymer paste, as well as improved adhesive properties to ceramics and faience. The compositions obtained have a high potential for application in the field of art, architecture, and design [45]. The authors of [46] investigated the possibility of using geopolymers to fill cracks, gaps, and voids in glazed ceramic tiles. The results of the studies showed that geopolymers have sufficient performance in bonding individual tiles and in the restoration of tiled facades.

Of great interest is the use of polyvinyl acetate (PVA) as an additive to geopolymer compositions. The geopolymerization reaction occurs in the alkaline medium of the activator solution, which, when polyvinyl acetate is added, causes its saponification reaction, followed by the decomposition of polyvinyl acetate into acetic acid and polyvinyl alcohol, which, together with the aluminosilicate binder, forms a stable composite structure [47]. In [48], the authors described the preparation and characteristics of a sustainable adhesive material for use in the field of art and design, consisting of a composite based on a geopolymer with PVA. The key idea of the research was to develop a material with reduced brittleness and increased adhesion to the most common surfaces in art and design. The microstructure of the adhesive composition developed by the authors was more homogeneous compared to pure geopolymer. A lower density (up to 15%) and increased bending strength (up to 30%) were observed. The new composition showed improved flow and adhesion to the most commonly used substrates, which allows it to be used for the restoration and restoration of sculptures and monuments, as well as in the field of decoration and architecture. This study is of interest from the point of view of the main components of the presented adhesive composition; however, the authors initially provide for rather small amounts of its application. The study [49] is devoted to the development of a geopolymer composite based on metakaolin and epoxy resin for use as a restoration composition. The resulting composition had consistency, workability, and thixotropic properties, which made it possible to effectively apply it to various substrates during the restoration, repair, and reinforcement of wall and ceiling surfaces.

This study develops the concept of sustainable building materials, which involves the replacement of traditional cement-based materials in construction and architecture with more environmentally friendly and sustainable alternatives based on geopolymers. Studies have been reviewed that have examined the effect of additives such as waste ceramics and PVA, but there is little information on the joint complex mechanism of the effect of these two components on the characteristics and structure of geopolymer composites used for the restoration of building facades. Therefore, the scientific novelty of this study is to obtain new dependencies of the characteristics and parameters of the structure of the geopolymer composite on the combined influence of additives of ceramic waste powder (PCW) and PVA. The scientific and practical novelty of the study is an experimentally substantiated and practically tested recipe for a new geopolymer concrete based on PCW and PVA. The work aimed to obtain geopolymer concrete with improved physical, mechanical, and adhesive characteristics, designed to restore the finishing of building facades. The objectives of the study were to study the properties of the main components of the geopolymer composite and the additives used, to check their compatibility; to obtain the dependences of the physical and mechanical characteristics of geopolymer concretes on the amount of PCW and PVA; to study the structural changes in geopolymer composites as a result of the complex influence of PCW and PVA; and to establish the possibility and expediency of using the developed compositions for work on the restoration of the facades of buildings and structures.

## 2. Materials and Methods

### 2.1. Materials

During the study, liquid glass Na_2_O(SiO_2_)n (Kubanzheldormash, Armavir, Russia) and NaOH (SUNTRADE, Lermontov, Russia) were used as an alkaline activator.

Metakaolin VMK-45 produced by Formako (Omsk, Russia) was used as the main binder. The chemical composition of metakaolin, expressed in oxide %, is presented in Table 1.

Quartz sand (Arkhipovsky quarry, Arkhipovskoye village, Russia), the characteristics of which are given in Table 2 (grain composition) and Table 3 (physical properties), was used in the study as a fine aggregate for the geopolymer composition.

As additives in geopolymer compositions, polyvinyl acetate (PVA) (Tikkurila, St. Petersburg, Russia) and powder from ceramic waste (PCW), obtained by mechanical processing of ceramic construction waste, consisting mainly of bricks, were used. Ceramic waste was crushed, dried to a stable weight at 105 °C, and sieved through a standard 2.5 mm sieve. After that, they were additionally crushed in an Activator-4M planetary ball mill (Chemical Engineering Plant, Novosibirsk, Russia) for 9 h at 700 rpm. The chemical composition of PCW, expressed in oxide %, is presented in Table 4.

The chemical composition of the ceramic waste powder was determined using a ZEISS CrossBeam 340 dual-beam scanning electron/ion microscope equipped with an Oxford Instruments X-Max 80 sensor microanalyzer (Jena, Germany). It can image and conduct a chemical analysis of samples.

**Figure 2:**
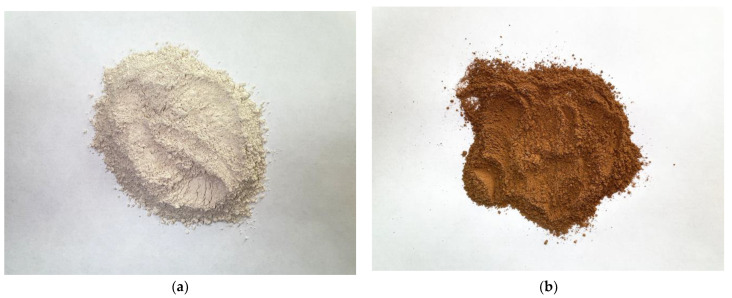
Photo of binder components: (**a**) metakaolin; (**b**) PCW.

Photos of the main components used as a binder to obtain a geopolymer composite are shown in Figure 2.

### 2.2. Methods

Samples of geopolymer concrete were made in laboratory conditions. Designs of experimental compositions of geopolymer concrete based on metakaolin with different contents of PCW and PVA are presented in Table 5.

The choice of proportions of geopolymer mixtures was based on the rational compositions of geopolymer concrete already selected by us in previous works [36,37,40] and on works of similar composition by other authors [45,48] with their adjustment related to the properties of the components used.

The alkaline activator solution was prepared by mixing 1:1 sodium hydroxide solution 6M and sodium silicate solution (SiO_2_ = 26.50%, Na_2_O = 8.70%, and pH = 11.8). NaOH solution was obtained under laboratory conditions by dissolving NaOH granules in distilled water to a concentration of 6M. The prepared solution of the alkaline activator was kept for 24 h before use.

The production of geopolymer mixtures and samples from them was carried out in the following sequence:

- dosing of all raw materials
- mixing of metakaolin, PCW, and sand
- introduction of an alkaline activator, liquid PVA into the resulting mixture and stirring the resulting mixture for 5 min
- filling of metal molds with geopolymer mixture
- vibrating the mixture in the molds for 10 s to remove unwanted air bubbles
- covering the forms with polyethylene film to protect against water evaporation for 24 h
- extraction of geopolymer concrete samples from molds after 24 h and storage for 27 days in plastic bags.

The main technological equipment used to produce geopolymer concrete is presented in Table 6.

**Figure 3:**
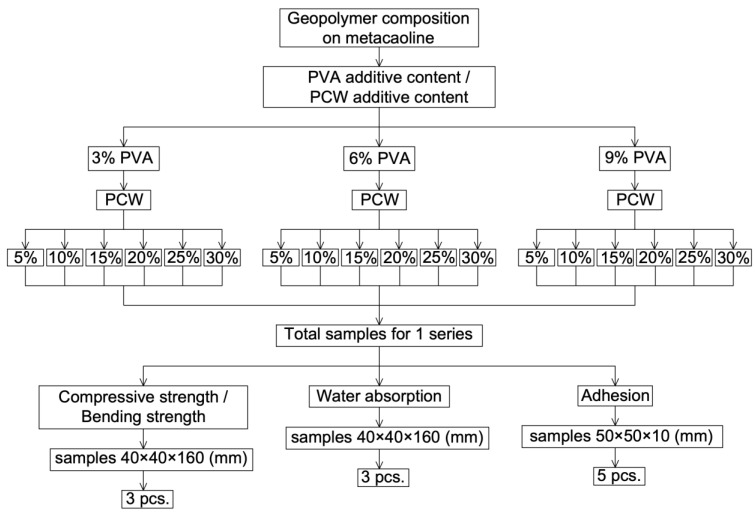
Test plan.

The test plan for samples of the hardened geopolymer mixture is shown in Figure 3.

During the study, standard methods for testing raw materials and products based on them were used.

Tests of the strength of specimens in compression and tension in bending were carried out in accordance with GOST 30744 [50]. Photos of testing samples are shown in Figure 3.

**Figure 4:**
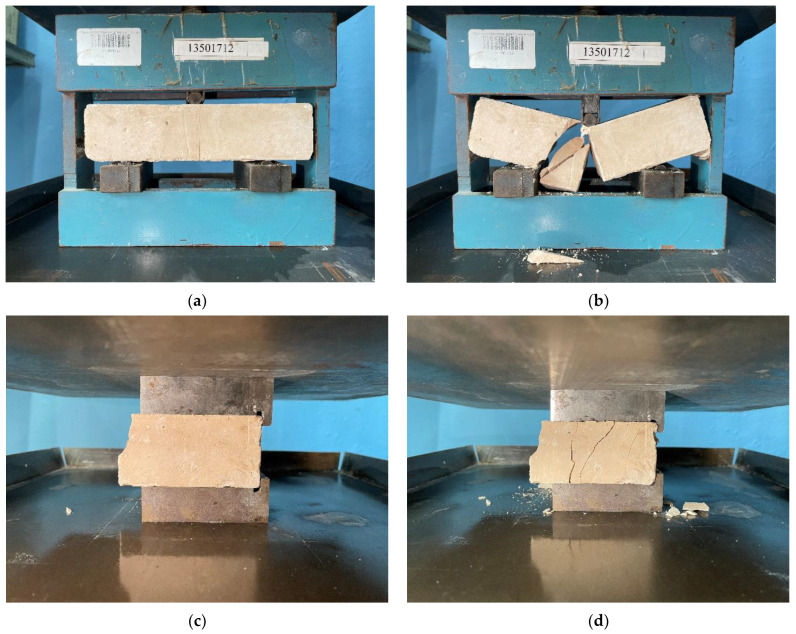
Testing specimens for bending strength (**a**,**b**) and compressive strength (**c**,**d**) before collapse and after collapse, respectively.

The sample was mounted on the support elements of the instrument (Figure 4a) in such a way that its faces, which were horizontal during manufacture, were in a vertical position. The average rate of increase in the load on the sample was (50 ± 10) N/s. The halves of beam specimens obtained after bending tests were immediately tested for compression. The sample half of the beam was placed between the pressure plates so that its faces, which were horizontal during manufacture, were in a vertical position (Figure 4b). In the longitudinal direction, the location of the halfway point of the sample beam should be such that its end protrudes from the pressure plates measuring 40 × 40 mm by about 10 mm. The bending strength *R*_btb_ (MPa) of a single sample was calculated using the formula:

(1) $$R_{\mathrm{btb}}=\frac{1.5F\;l}{b^{3}}$$

where *F* is the breaking load (*N*), *b* is the size of the side of the square section of the sample (mm), *l* is the distance between the axes of the supports (mm).

The arithmetic mean of the test results for three specimens was taken as the bending strength. The calculation result was rounded up to 0.1 MPa.

The compressive strength *R*_b_ (MPa) of an individual half of the sample after the bending test was calculated by the formula:

(2) $$R_{b}=\frac{F}{S}$$

where *F* is the breaking load (N), and *S* is the area of the working surface of the pressure plate (mm^2^).

For the compressive strength, the arithmetic average of the test results of six halves of the samples obtained after the bending test was taken. The calculation result was rounded up to 0.1 MPa.

Water absorption tests of the geopolymer composite with the addition of PWC and PVA were carried out in accordance with GOST 12730.3 [51]. During the water absorption tests, the samples were placed in a container filled with water, on gaskets so that their height was minimal and the water level in the container was 50 mm higher than the surface of the samples. The water temperature was 20 ± 2 °C. Every 24 h, the samples were taken out of the container, wiped with a wrung-out damp cloth, and weighed on a conventional balance with an error of less than 0.1% until the results of two consecutive weighings began to differ by less than 0.1%. Next, the samples were dried to a constant weight in an ShS-80-01 SPU drying oven (Smolensk SKTB SPU, Smolensk, Russia). Furthermore, according to the test results, the water absorption of concrete of each sample was determined by weight with an error of up to 0.01%. The water absorption of concrete of each sample *W_m_* (% wt.) was calculated with an error of up to 0.1% according to the formula:

(3) $$W_{m}=\frac{m_{w}-m_{d}}{m_{d}}$$

where *m_w_* is the mass of the sample saturated with water (g), and *m_d_* is the mass of the dried sample (g).

Tests to determine the adhesion strength (adhesion) of a geopolymer composite with a base of concrete and ceramic (brick) were carried out in accordance with GOST R 58,277 [52]. The test specimens were made in the form of prisms with a square cross-section of 50 × 50 mm and a thickness of 10 mm. A stencil was installed on a concrete or ceramic base, on which the mixture was applied and smoothed with a metal spatula, after which the stencil was immediately removed. After 28 days, a stamp was glued to the hardened samples with Moment high-strength epoxy adhesive (Henkel, Düsseldorf, Germany), and the samples were stored at a temperature of (20 ± 2) °C and relative humidity (65 ± 5)% for 24 h. The test was carried out using an adhesive meter until the composite sample is detached from the base. The force of detachment of the samples from the base was determined by applying a force to the stamp at a rate of its increase (250 ± 50) N/s. The adhesion strength (adhesion) of the sample to the base was determined as the maximum force applied perpendicular to the surface of the sample, at which the sample detached from the base. Adhesion strength (adhesion) with the base when testing one sample Ai (MPa) was calculated by the formula:

(4) $$A_{i}=\frac{F}{S}$$

where *F* is the maximum force of detachment of the sample from the base (N), and *S* is the contact area of the sample surface with the base (mm^2^).

The result was taken as the arithmetic mean of the test results of all samples A (MPa), calculated by the formula:

(5) $$A=\frac{A_{1}+\ldots +A_{5}}{5}$$

When determining the above characteristics of geopolymer compositions, the following test equipment was used: Oniks-1.AP adhesive meter (Interpribor, Chelyabinsk, Russia); press IP-1000 (NPK TEHMASH, Neftekamsk, Russia).

**Figure 5:**
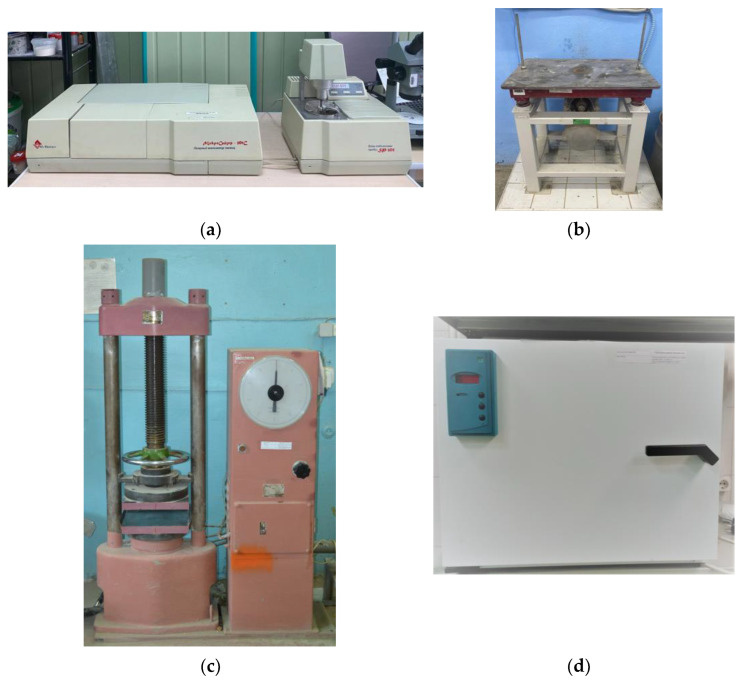
The main equipment used in the study: (**a**) Microsizer 201C for particle size analysis; (**b**) laboratory vibration platform for vibrating the mixture in the molds; (**c**) strength test press; (**d**) water absorption test oven.

Particle size analysis was performed on a Microsizer 201C instrument (VA Insalt, St. Petersburg, Russia). Microscopic studies were carried out on a ZEISS CrossBeam 340 microscope (Carl Zeiss Microscopy GMBH (Factory), Jena, Germany) with magnifications of 100 and 1000 times. Chemical analysis was carried out using the Oxford Instruments X-Max 80 microanalyzer, which is equipped with the ZEISS CrossBeam 340. Photos of the main equipment used in the study are shown in Figure 5.

## 3. Results

### 3.1. Particle Distribution Study of Metakaolin and PCW

**Figure 6:**
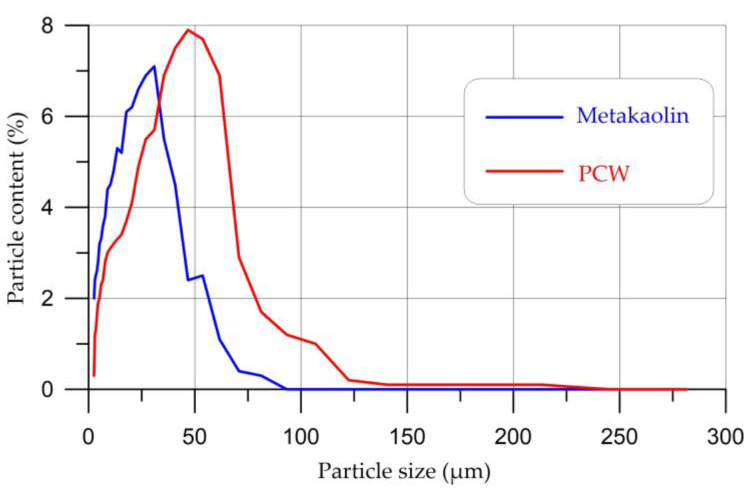
Particle distribution curves of metakaolin and PCW.

An analysis of the particle sizes of metakaolin and PCW is presented in Figure 6.

Based on the results of the analysis of metakaolin and PCW particles, the following was established. The largest part of metakaolin particles (93.3%) is in the range from 2 to 45 µm, the distribution peak falls on particles with a size of 30 µm and is 7.1%. As for PCW particles, the largest part of them (92.6%) is in the size range from 2 to 70 µm, the distribution peak falls on particles with a size of 46 µm and is 7.9%.

### 3.2. Study of the Influence of PCW and PVA on the Strength Characteristics of Geopolymer Composites

**Figure 7:**
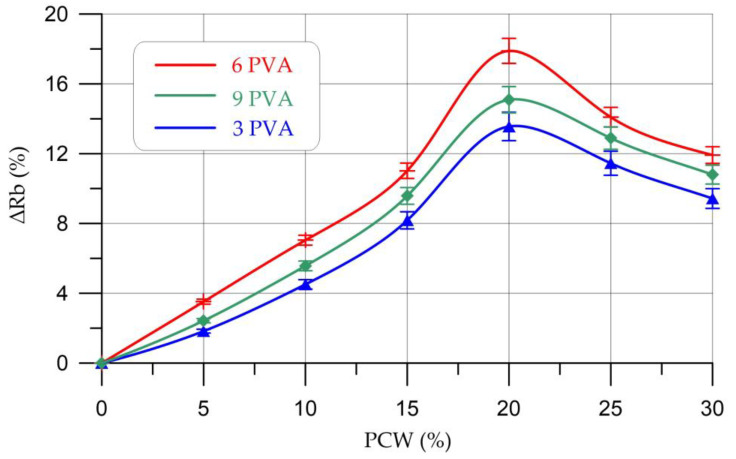
Changes in compressive strength (∆*R*_b_) of geopolymer composites depending on the content of PCW and PVA.

**Figure 8:**
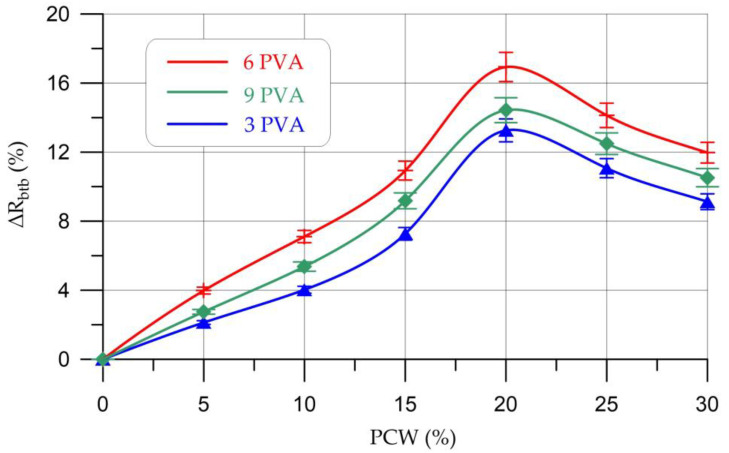
Change in bending strength (∆*R*_btb_) of geopolymer composites depending on the content of PCW and PVA.

The results of determining the strength characteristics of geopolymer compositions based on metakaolin with PCW and PVA additives are presented in Table 7 and Figure 7 and Figure 8.

Figure 7 shows that the use of ceramic waste powder and PVA additive has a positive effect on the compressive strength of geopolymer concrete based on metakaolin. Based on the obtained values, the best increases in compressive strength for all compositions are observed with partial replacement of PCW metakaolin in an amount of 20%. In addition, for a PVA supplement, the most effective dosage is 6%. In general, a positive trend in the change in the compressive strength of geopolymer concretes is observed when the content of the PCW additive is from 5% to 20% inclusive, and at 25–30%, the compressive strength begins to gradually decrease. Thus, a further increase in the PCW content will adversely affect the compressive strength values. When a part of metakaolin was replaced with a PCW additive in an amount of 20% and with a PVA additive content in an amount of 6%, the maximum increase in compressive strength was recorded, which amounted to 17.89%.

The nature of the change in bending strength is similar to the change in compressive strength, i.e., an increase in bending strength is observed at a PCW content of 5–20% and a decrease, starting from 25% PCW. The maximum increase in bending strength was also recorded at a PCW content of 20% and PVA of 6% and amounted to 16.93%. The increase in strength is due to the good dispersion of PCW in the geopolymer composite, and thus the creation of barriers against crack growth [45]. At the same time, the addition of PVA also contributes to a more compact and homogeneous structure of the composite with a reduced number of voids compared to the control composition of the geopolymer [48]. When the content of PCW is more than 20%, a decrease in strength characteristics is observed, which is explained by a violation of the connectivity of the structure of the geopolymer composite and the formation of a larger number of pores and microcracks. Additionally, when the content of PCW is more than 20%, the content of unreacted particles increases due to a decrease in the proportion of the main binder component [40,45].

### 3.3. Study of the Effect of PCW and PVA on the Water Absorption of Geopolymer Composites

**Figure 9:**
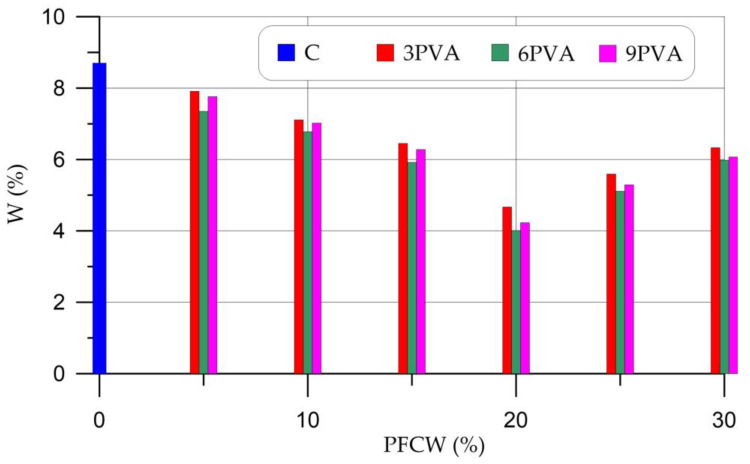
Water absorption of samples of geopolymer concretes with different percentages of replacement of metakaolin with PCW and different dosages of PVA.

Figure 9 shows the results of determining the water absorption of samples of geopolymer concretes with different percentages of replacement of metakaolin by PCW and different dosages of PVA.

Figure 9 shows that samples with a PVA content of 6% and PCW of 20% have the lowest water absorption, up to 54% less than that of the control composition. The general nature of the change in water absorption can be described as follows: a decrease in water absorption with the replacement of a part of metakaolin with 5–20% PCW and, conversely, an increase in water absorption with the replacement of a part of metakaolin with 25–30% PCW. At any percentage of PCW replacement, formulations containing 6% PVA additive show the best values. With a PVA content of 6%, geopolymers have the best thixotropy. This means that all compositions with a given dosage of PVA will have less porosity and less water absorption, regardless of the considered dosages of PCW. The decrease in water absorption of modified PCW and PVA compositions from geopolymer concrete is explained by better cohesion of the structure, which contributes to an increase in strength by reducing defects and creating barriers to the formation and development of cracks and voids in the composite [45,47]. The presence of high adhesion between the newly formed phases and unreacted particles of the aluminosilicate component ensured high strength and low water absorption of the hardened geopolymer composite [40].

### 3.4. Study of the Effect of PCW and PVA on the Adhesion of Geopolymer Composites

**Figure 10:**
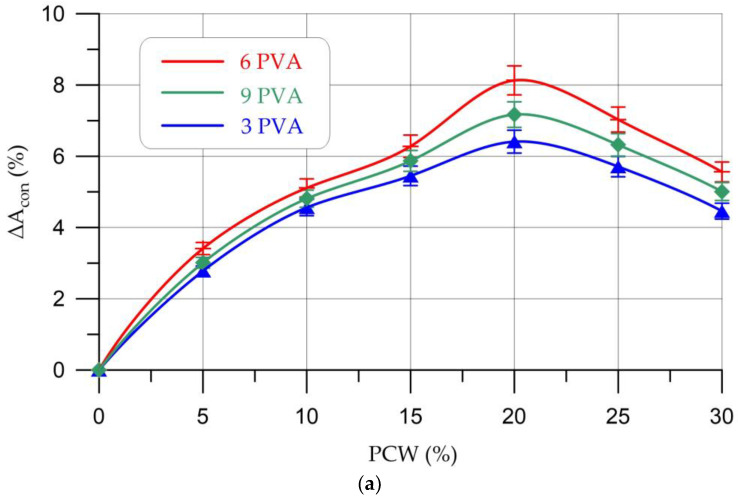

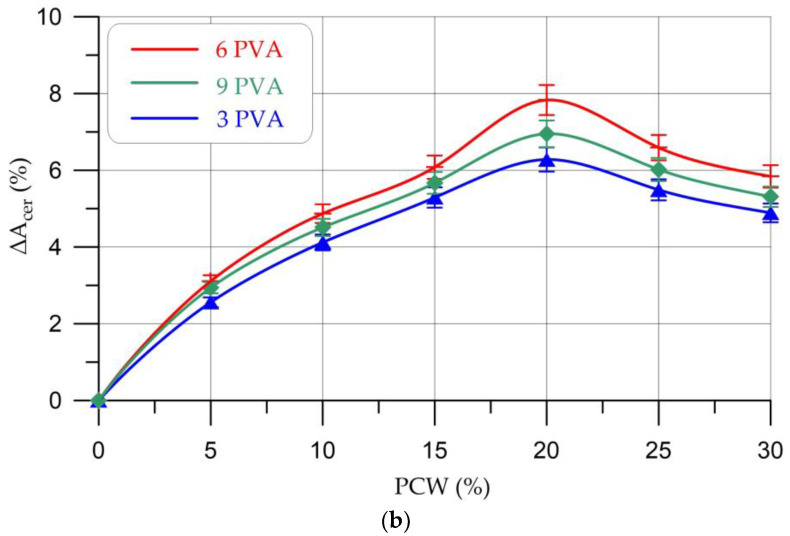
Change in adhesion of geopolymer composites (**a**) with a concrete base; (**b**) with ceramic base depending on PCW and PVA content.

Table 8 presents the results of determining the adhesion of experimental compositions of geopolymer concretes with a concrete and ceramic base, and Figure 10 shows the dependence of the adhesion strength (adhesion) of geopolymer composites on the content of PCW and PVA.

Figure 10 shows that, as in the case of strength characteristics and water absorption, the most effective composition is PVA content of 6% and PCW of 20%. The adhesion trend is as follows: an increase at a dosage of PCW from 5% to 20% (8.3% more than the control composition for a concrete base and 7.8% more for a ceramic base) and a decrease at dosages of 25–30%. At the same time, adhesion with a concrete base is slightly higher (up to 5%) than with a ceramic base. This can be explained by the rougher surface of the concrete base compared to the ceramic one. As is known, the main factors affecting the adhesion strength of geopolymer concretes are the friction force and chemical bonds. At the macro level, the composition of geopolymer concrete envelops the bonded surface with an uneven base, creating friction forces against each other. At the micro level, a dense interfacial transition zone between fine aggregate and geopolymer paste gives the geopolymer high adhesive strength [16]. Accordingly, the introduction of the PVA additive into the geopolymer mixture in a rational dosage (6%) helps to increase the adhesion between the geopolymer paste and the aggregate, which in turn increases the adhesive properties of the geopolymer concrete.

### 3.5. Analysis of the Microstructure of Geopolymer Concrete with the Addition of PCW and PVA

**Figure 11:**
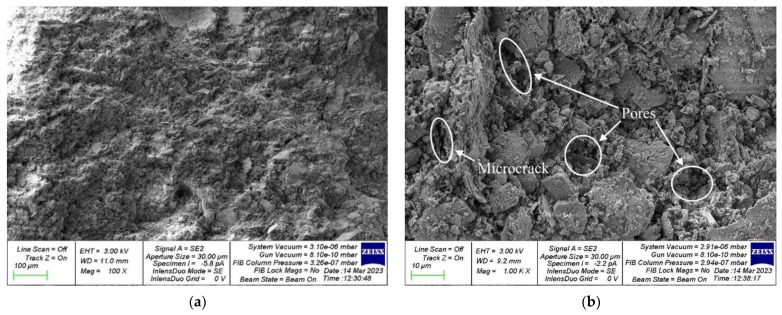
A sample of geopolymer concrete of the control composition: (**a**) 100×; (**b**) 1000×.

**Figure 12:**
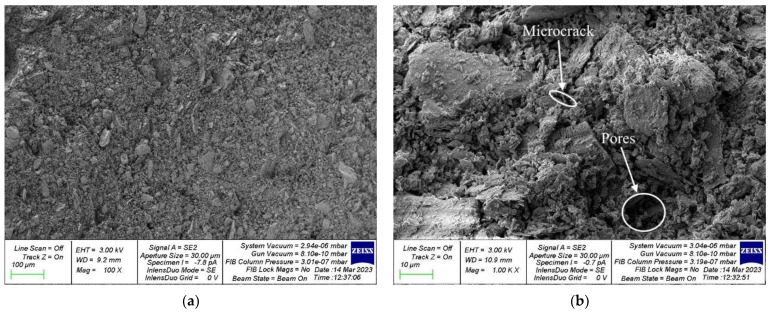
A sample of geopolymer concrete of composition type 4A/6: (**a**) 100×; (**b**) 1000×.

The study of the microstructure was carried out to confirm the obtained dependencies between the physical and mechanical characteristics of geopolymer concrete and dosages of PCW and PVA and to compare the structures of geopolymer concrete of the control composition and composition with a rational dosage of PCW and PVA at the micro level. For the study, samples of geopolymer concrete of the control composition and composition of type 4A/6 were selected with 20% PFCW additive introduced instead of a part of metakaolin, and 6% PVA additive. Images of the microstructure of the samples of the hardened geopolymer composite of the control composition and the composition with the most effective dosages of PCW and PVA additives are shown in Figure 11 and Figure 12, respectively.

As can be seen, geopolymer concrete composition type 4A/6 (Figure 12) has the densest structure with fewer pores and microcracks compared to the control composition of geopolymer concrete (Figure 11). This is because PCW particles, brought to the state of a finely dispersed powder, play the role of a micromodifier, which contributes to the creation of additional crystallization centers, as a result of which structure formation is improved, and a denser packing of particles occurs. Thus, the composition 4A/6 has a denser structure and a more perfect homogenization process and, thus, all this contributes to a better technology for hardening such geopolymer concrete, improved physical and chemical processes of structure formation occur and, at the same time, mechanical properties that are directly dependent on structures also increase. Thus, the use of PCW performs not only an ecological role but also contributes to the improvement of quality and the creation of highly functional geopolymer concretes. The addition of PVA to the geopolymer mixture helps to reduce the number of pores and microcracks, which in turn leads to an increase in bending strength and a decrease in water absorption. In an alkaline environment in which the geopolymerization reaction occurs, PVA hydrolyzes to a mixed polymer containing both hydroxyl and acetyl groups. Due to the good water solubility of PVA, no isolated areas of its concentration are observed in the microstructure of the geopolymer composite, and it is evenly distributed in the mixture. Additionally, the addition of PVA in an amount of 6% by weight of the binder leads to an improvement in the thixotropy of the composition and an increase in adhesion [16,40,45,48].

## 4. Discussion

The results obtained in this study are in good agreement with the results obtained earlier by other authors [9,39,45,46,48,49,53,54] in terms of improving the physical and mechanical characteristics due to the addition of PCW and PVA. For example, in [9,53], the addition of ceramic wastes to geopolymer composites based on fly ash and ground granulated blast-furnace slag made it possible to achieve an increase in such characteristics as compressive and flexural strength, as well as frost resistance and durability, which is in good agreement with a decrease in water absorption of improved geopolymer composites in this study. In the study [54], geopolymer composites containing powder from ceramic waste in an amount of 50–60% in the total share of the binder showed the best values of abrasion resistance, as well as better frost resistance and lower water absorption values, which significantly differs from the value of the rational dosage obtained in current work. The dosage of 5% PVA showed the highest efficiency in the geopolymer composite [48], which is in good agreement with the obtained rational dosage of PVA in this study (6%). Summing up the above-analyzed studies, the following can be noted. The use of PCW and PVA in the technology of geopolymer concretes made from various types of aluminosilicate base, with a selected rational dosage of components, increases the strength of composites and improves long-term properties. In addition, the studies carried out in this work confirm the effectiveness of the combined use of PCW and PVA in the composition of geopolymer concretes based on metakaolin to restore the finishing of facades of buildings and structures.

The analysis of the results obtained and explanation of the mechanism for the formation of an improved structure and properties of geopolymer concrete depending on its composition is as follows. From the point of view of improving the structure at the micro- and macrolevels, the high effect achieved is explained by the fact that the introduced PCW additive is a modifier of the existing geopolymer composition, making it possible to obtain a fundamentally new geopolymer concrete with improved physical-mechanical and, first of all, adhesive characteristics. Such an increased adhesive ability is explained by a more developed surface of the obtained particles, and in the process of formation of the structure and hardening of geopolymer concretes, an additional effect arises in terms of better adhesion of the geopolymer composite to the base for plaster mortar. The developed surface of the particles, their fineness, as well as the good compatibility of PCW with other components of geopolymer concrete, make it possible to achieve better operational reliability of the new composition. At the level of formation of properties, depending on the improved structure already obtained, such particles, in addition to the developed surface, also make it possible to achieve the appearance of additional crystallization centers during structure formation, which contributes to the aggregation of geopolymer concrete particles around the modifier particles, which is well confirmed not only by the high results of physical and mechanical tests but also by SEM analysis. Together, the improvement of the structure at the micro- and macrolevels, as well as the physical-mechanical and adhesive properties with a developed surface of new geopolymer concretes, allow us to offer the developed compositions for practical work on the restoration of the facades of buildings and structures, including those operated in difficult conditions due to improved quality indicators.

In general, the experiments have shown that the characteristics of the compositions of geopolymer concrete from metakaolin with PCW and PVA make it possible to effectively use them for restoring the finishing of building facades. The most effective for use in this area is the composition type 4A/6, which showed the best characteristics of compressive strength, flexural strength, water absorption, and adhesion. Geopolymer concrete compositions based on metakaolin with the addition of PCW and PVA can be used to eliminate facade defects spread over large areas, as a finishing material, to restore lost parts of complex facade elements, as a composition for filling and sealing cracks in brickwork, porcelain stoneware, and natural stone. However, to fully restore not only the operational but also the aesthetic properties of the facades of buildings, it is necessary to provide a color and texture similar to those of the restored elements. At the same time, the existing restoration practice suggests that the composition of geopolymer concrete should be well distinguishable from the surface being restored to enable its complete removal without damaging the original product. To ensure these conditions, it is necessary to observe a slight difference in the color of the concrete from the color of the restored surface, for example, a little lighter or a little darker. The resulting rational composition based on geopolymer concrete with the addition of ceramic waste and PVA can be easily colored with water-based pigments by adding them to the mixture while mixing the components. This method provides a uniform coloring of the hardened composition throughout the volume. In the case when it is necessary to create a complexly colored surface, the resulting composition provides the possibility of painting its surface after hardening with oil paints or cold painting using complex techniques that allow you to completely imitate the restored surface. A more detailed imitation of the texture of the restored surface can be achieved by adding a powder of the material from which the restored element is made.

The resulting geopolymer concrete, using waste as a component, can provide environmental savings of up to 17% compared to existing concrete compositions. These savings are estimated based on approximate feedback from industrial partners and are due to data on the disposal of broken bricks generated in construction. In addition, the second environmental effect is the saving of cement, and, consequently, a decrease in the need for its produced quantity. This leads to better environmental conditions and lower CO_2_ emissions.

## 5. Conclusions

According to the results of the studies of geopolymer concretes, the following conclusions can be drawn.

(1) The most optimal dosages of PCW and PVA additives have been established, at which geopolymer concretes have the best physical, mechanical, and structural characteristics: 20% PCW introduced into the geopolymer instead of a part of metakaolin, and 6% PVA.
(2) The combined use of PCW and PVA additives in optimal dosages provides the maximum increase in strength and physical characteristics. Compressive strength increased by up to 18%, bending strength up to 17%, water absorption of geopolymer concretes decreased by up to 54%, and adhesion increased by up to 9%. The increase in strength is due to the good dispersion of PCW in the geopolymer composite and thus the creation of barriers against crack growth. The addition of PVA also contributes to a more compact and homogeneous structure of the composite with a reduced number of voids compared to the control composition of the geopolymer. The adhesion of the modified geopolymer composite is slightly better with a concrete base than with a ceramic one (up to 5%) due to the more developed rough surface of the first one in comparison with a smooth ceramic one.
(3) Geopolymer concretes modified with PCW and PVA additives have a denser structure with fewer pores and microcracks due to the presence of PCW brought to the state of a fine powder, playing the role of a micromodifier, which contributes to the creation of additional crystallization centers, as a result of which structure formation improves and occurs.
(4) The application of PCW in combination with a cementless binder provides environmental savings of up to 17% compared to existing cement-based formulations, estimated based on rough feedback from industry partners and data on the disposal of broken bricks generated during construction.
(5) Improving the structure at the micro- and macrolevels, as well as the physical-mechanical and adhesive properties with a developed surface of new geopolymer concretes, allow us to offer the developed compositions for practical work on the restoration of the facades of buildings and structures, including those operated in difficult conditions due to improved quality indicators.

Prospects for continuing research lie in the direction of studying other types of additives that increase the adhesive and other operational reliability of plaster compositions, including those for the restoration of unique buildings and facades of historical cultural heritage sites.

## Figures and Tables

**Table 1:** Chemical composition of metakaolin.

Oxide	Content (%)
SiO_2_	53.79
Al_2_O_3_	40.03
Fe_2_O_3_	1.45
MgO	0.34
CaO	0.14
Na_2_O	0.09
K_2_O	0.69
TiO_2_	2.21
P_2_O_5_	0.05
LOI	1.21

The chemical composition of metakaolin was provided by the manufacturer.

**Table 2:** Grain composition and modulus of sand size.

Residue on Sieves (%)	Sieves Diameter (mm)						Size Modulus
	2.5	1.25	0.63	0.315	0.16	<0.16	
Partial	1.28	1.28	10.51	45.04	39.74	2.15	1.73
Full	1.28	2.56	13.07	58.11	97.85		

**Table 3:** Physical properties of sand.

Bulk density (kg/m^3^)	1578
True density (kg/m^3^)	2675
The content of dust and clay particles (%)	1.1
Clay content in lumps (%)	0.15
Content of organic and contaminants	-

**Table 4:** Chemical composition of PCW.

Oxide	Content (%)
SiO_2_	49.2
Al_2_O_3_	20.3
CaO	17.3
Fe_2_O_3_	4.4
K_2_O	2.2
MgO	1.5
Na_2_O	1.4
TiO_2_	0.5
Others	3.2

**Table 5:** Proportions of geopolymer mixtures based on metakaolin.

Sample Code	The Ratio of Alkaline Activator/ Binder	The Ratio of Binder/ Aggregate	Binder Metakaolin + PCW (%)	PVA Content (wt. % of Binder)
K	0.5	1.5	100 + 0	3
1A	0.5	1.5	95 + 5	3
2A	0.5	1.5	90 + 10	3
3A	0.5	1.5	85 + 15	3
4A	0.5	1.5	80 + 20	3
5A	0.5	1.5	75 + 25	3
6A	0.5	1.5	70 + 30	3
7A	0.5	1.5	95 + 5	6
8A	0.5	1.5	90 + 10	6
9A	0.5	1.5	85 + 15	6
10A	0.5	1.5	80 + 20	6
11A	0.5	1.5	75 + 25	6
12A	0.5	1.5	70 + 30	6
13A	0.5	1.5	95 + 5	9
14A	0.5	1.5	90 + 10	9
15A	0.5	1.5	85 + 15	9
16A	0.5	1.5	80 + 20	9
17A	0.5	1.5	75 + 25	9
18A	0.5	1.5	70 + 30	9

**Table 6:** Technological equipment.

Technological Operation	Equipment
Dosing and mixing of components	Laboratory concrete mixer BL-10 (ZZBO LLC, Russia, Chelyabinsk region, Zlatoust)
Sample making	Laboratory vibration platform SMZh-539-220A (IMASH, Armavir, Russia)

**Table 7:** Results of determining the strength characteristics of geopolymer compositions.

Sample Code	Compressive Strength *R*_b_ (MPa)	Flexural Strength *R*_btb_ (MPa)
C	18.7	2.70
1A/3	19.0	2.76
2A/3	19.5	2.81
3A/3	20.2	2.90
4A/3	21.2	3.06
5A/3	20.8	3.00
6A/3	20.4	2.95
1A/6	19.3	2.81
2A/6	20.0	2.89
3A/6	20.7	3.00
4A/6	22.0	3.16
5A/6	21.3	3.08
6A/6	20.9	3.02
1A/9	19.1	2.77
2A/9	19.7	2.84
3A/9	20.5	2.95
4A/9	21.5	3.09
5A/9	21.1	3.04
6A/9	20.7	2.98

**Table 8:** Adhesion of geopolymer composites.

Sample Code	Adhesion Strength with Concrete Base (Adhesion), MPa	Adhesion Strength with Ceramic Base (Adhesion) (MPa)
C	0.548	0.460
1A/3	0.563	0.472
2A/3	0.573	0.479
3A/3	0.578	0.484
4A/3	0.583	0.489
5A/3	0.579	0.485
6A/3	0.572	0.482
1A/6	0.567	0.474
2A/6	0.576	0.482
3A/6	0.582	0.488
4A/6	0.593	0.496
5A/6	0.587	0.490
6A/6	0.578	0.487
1A/9	0.564	0.474
2A/9	0.574	0.481
3A/9	0.580	0.486
4A/9	0.587	0.492
5A/9	0.583	0.488
6A/9	0.575	0.484

## Data Availability

The study did not report any data.

## References

[1] Khan K., Ahmad W., Amin M.N., Nazar S. (2022). A Scientometric-Analysis-Based Review of the Research Development on Geopolymers. Polymers.

[2] Castillo H., Collado H., Droguett T., Vesely M., Garrido P., Palma S. (2022). State of the art of geopolymers: A review. e-Polymers.

[3] Dollente I.J.R., Valerio D.N.R., Quiatchon P.R.J., Abulencia A.B., Villoria M.B.D., Garciano L.E.O., Promentilla M.A.B., Guades E.J., Ongpeng J.M.C. (2023). Enhancing the Mechanical Properties of Historical Masonry Using Fiber-Reinforced Geopolymers. Polymers.

[4] Aliabdo A.A., Elmoaty A.E.M.A., Emam M.A. (2019). Factors affecting the mechanical properties of alkali activated ground granulated blast furnace slag concrete. Constr. Build. Mater..

[5] McCaffrey R. (2002). Climate change and the cement industry. Glob. Cem. Lime Mag. (Environ. Spec. Issue).

[6] WMO (2019). Greenhouse Gas. Bulletin: The State of the Greenhouse Gases in the Atmosphere Based on Global Observations through 2018. Weather Clim. Water.

[7] Rashad A.M. (2013). A comprehensive overview about the influence of different additives on the properties of alkali-activated slag–A guide for civil engineer. Constr. Build. Mater..

[8] Davidovits J. (2017). Geopolymers: Ceramic-Like Inorganic Polymers. J. Ceram. Sci. Technol..

[9] Huseien G.F., Kubba Z., Mhaya A.M., Malik N.H., Mirza J. (2023). Impact Resistance Enhancement of Sustainable Geopolymer Composites Using High Volume Tile Ceramic Wastes. J. Compos. Sci..

[10] Dal Poggetto G., D’Angelo A., Catauro M., Barbieri L., Leonelli C. (2022). Recycling of Waste Corundum Abrasive Powder in MK-Based Geopolymers. Polymers.

[11] Azad N.M., Samarakoon S.M.S.M.K. (2021). Utilization of Industrial By-Products/Waste to Manufacture Geopolymer Cement/Concrete. Sustainability.

[12] Bhikshma V., Koti R.M., Srinivas R.T. (2012). An experimental investigation on properties of geopolymer concrete (no cement concrete). Asian J. Civ. Eng. (Build. Hous.).

[13] Bhina M.R., Liu K.-Y., Hu J.-E.H.-Y., Tsai C.-T. (2023). Investigation of the Mechanical Properties of Quick-Strength Geopolymer Material Considering Preheated-to-Room Temperature Ratio of Sand, Na_2_SiO_3_-to-NaOH Ratio, and Fly Ash-to-GGBS Ratio. Polymers.

[14] Alyousef R., Ebid A.A.K., Huseien G.F., Mohammadhosseini H., Alabduljabbar H., Poi Ngian S., Mohamed A.M. (2022). Effects of Sulfate and Sulfuric Acid on Efficiency of Geopolymers as Concrete Repair Materials. Gels.

[15] Ozga I., Ghedini N., Giosuè C., Sabbioni C., Tittarelli F., Bonazza A. (2014). Assessment of air pollutant sources in the deposit on monuments by multivariate analysis. Sci. Total Environ..

[16] Feng B., Liu J. (2022). Durability of Repair Metakaolin Geopolymeric Cement under Different Factors. Processes.

[17] Hanzlícek T., Steinerová M., Straka P., Perná I., Siegl P., Švarcová T. (2009). Reinforcement of the terracotta sculpture by geopolymer composite. Mater. Des..

[18] Eken E., Tascı B., Gustafsson C. (2019). An evaluation of decision-making process on maintenance of built cultural heritage: The case of Visby, Sweden. Cities.

[19] Beskopylny A.N., Stel’makh S.A., Shcherban’ E.M., Mailyan L.R., Meskhi B., Beskopylny N., El’shaeva D., Kotenko M. (2022). The Investigation of Compacting Cement Systems for Studying the Fundamental Process of Cement Gel Formation. Gels.

[20] Kim J., Lee D., Sičáková A., Kim N. (2023). Utilization of Different Forms of Demolished Clay Brick and Granite Wastes for Better Performance in Cement Composites. Buildings.

[21] Provis J.L. (2014). Green concrete or red herring?—Future of alkali-activated materials. Adv. Appl. Ceram..

[22] Barbhuiya S., Pang E. (2022). Strength and Microstructure of Geopolymer Based on Fly Ash and Metakaolin. Materials.

[23] Jihui Z. (2021). Eco-friendly geopolymer materials: A review of performance improvement, potential application and sustainability assessment. J. Clean. Prod..

[24] Occhicone A., Vukčević M., Bosković I., Mingione S., Ferone C. (2022). Alkali-Activated Red Mud and Construction and Demolition Waste-Based Components: Characterization and Environmental Assessment. Materials.

[25] Korniejenko K., Łach M., Mikuła J. (2021). The Influence of Short Coir, Glass and Carbon Fibers on the Properties of Composites with Geopolymer Matrix. Materials.

[26] Pacheco-Torgal F., Jalali S. (2021). Retraction Note: Compressive strength and durability properties of ceramic wastes based concrete. Mater. Struct..

[27] Silva T.H., de Resende M.C., de Resende D.S., Ribeiro Soares P.R., da Silva Bezerra A.C. (2022). Valorization of ceramic sludge waste as alternative flux: A way to clean production in the sanitary ware industry. Clean. Eng. Technol..

[28] Luhar I., Luhar S., Abdullah M.M.A.B., Nabiałek M., Sandu A.V., Szmidla J., Jurczyńska A., Razak R.A., Aziz I.H.A., Jamil N.H. (2021). Assessment of the Suitability of Ceramic Waste in Geopolymer Composites: An Appraisal. Materials.

[29] Ramos G.A., de Matos P.R., Pelisser F., Gleize P.J.P. (2020). Effect of porcelain tile polishing residue on eco-efficient geopolymer: Rheological performance of pastes and mortars. J. Build. Eng..

[30] Zeyad A.M., Magbool H.M., Tayeh B.A., de Azevedo AR G., Abutaleb A., Hussain Q. (2022). Production of geopolymer concrete by utilizing volcanic pumice dust. Case Stud. Constr. Mater..

[31] Nasaeng P., Wongsa A., Cheerarot R., Sata V., Chindaprasirt P. (2022). Strength enhancement of pumice-based geopolymer paste by incorporating recycled concrete and calcined oyster shell powders. Case Stud. Constr. Mater..

[32] Occhicone A., Vukčević M., Bosković I., Ferone C. (2021). Red mud-blast furnace slag-based alkali-activated materials. Sustainability.

[33] Roviello G., Ricciotti L., Molino A.J., Menna C., Ferone C., Asprone D., Cioffi R., Ferrandiz-Mas V., Russo P., Tarallo O. (2020). Hybrid fly ash-based geopolymeric foams: Microstructural, thermal and mechanical properties. Materials.

[34] Botti R.F., Innocentini M.D.M., Faleiros T.A., Mello M.F., Flumignan D.L., Santos L.K., Franchin G., Colombo P. (2020). Biodiesel Processing Using Sodium and Potassium Geopolymer Powders as Heterogeneous Catalysts. Molecules.

[35] Kozhukhova N.I., Fomina E.V., Zhernovsky I.V., Strokova V.V., Chizhov R.V. (2014). The Utilization Efficiency of Natural Alumosilicates in Composite Binders. Appl. Mech. Mater..

[36] Shcherban’ E.M., Stel’makh S.A., Beskopylny A., Mailyan L.R., Meskhi B. (2022). Increasing the Corrosion Resistance and Durability of Geopolymer Concrete Structures of Agricultural Buildings Operating in Specific Conditions of Aggressive Environments of Livestock Buildings. Appl. Sci..

[37] Beskopylny A.N., Stel’makh S.A., Shcherban’ E.M., Mailyan L.R., Meskhi B., El’shaeva D., Varavka V. (2021). Developing Environmentally Sustainable and Cost-Effective Geopolymer Concrete with Improved Characteristics. Sustainability.

[38] Han W., Lv Y., Wang S., Qiao J., Zou C., Su M., Peng H. (2023). Effects of Al/Na and Si/Na Molar Ratios on the Alkalinity of Metakaolin-Based Geopolymer Pore Solutions. Materials.

[39] Liu X., Liu E., Fu Y. (2023). Reduction in Drying Shrinkage and Efflorescence of Recycled Brick and Concrete Fine Powder–Slag-Based Geopolymer. Appl. Sci..

[40] Beskopylny A.N., Shcherban’ E.M., Stel’makh S.A., Mailyan L.R., Meskhi B., El’shaeva D. (2022). The Influence of Composition and Recipe Dosage on the Strength Characteristics of New Geopolymer Concrete with the Use of Stone Flour. Appl. Sci..

[41] Beskopylny A.N., Stel’makh S.A., Shcherban’ E.M., Mailyan L.R., Meskhi B., Varavka V., Beskopylny N., El’shaeva D. (2022). A Study on the Cement Gel Formation Process during the Creation of Nanomodified High-Performance Concrete Based on Nanosilica. Gels.

[42] Stel’makh S.A., Shcherban’ E.M., Beskopylny A., Mailyan L.R., Meskhi B., Beskopylny N., Zherebtsov Y. (2022). Development of High-Tech Self-Compacting Concrete Mixtures Based on Nano-Modifiers of Various Types. Materials.

[43] Beskopylny A.N., Stel’makh S.A., Shcherban’ E.M., Mailyan L.R., Meskhi B., Shilov A.A., Chernil’nik A., El’shaeva D. (2023). Effect of Walnut-Shell Additive on the Structure and Characteristics of Concrete. Materials.

[44] Beskopylny A.N., Stel’makh S.A., Shcherban’ E.M., Mailyan L.R., Meskhi B., Smolyanichenko A.S., Beskopylny N. (2022). High-Performance Concrete Nanomodified with Recycled Rice Straw Biochar. Appl. Sci..

[45] Ricciotti L., Occhicone A., Ferone C., Cioffi R., Tarallo O., Roviello G. (2022). Development of Geopolymer-Based Materials with Ceramic Waste for Artistic and Restoration Applications. Materials.

[46] Moutinho S., Costa C., Andrejkovičová S., Mariz L., Sequeira C., Terroso D., Rocha F., Velosa A. (2020). Assessment of properties of metakaolin-based geopolymers applied in the conservation of tile facades. Constr. Build. Mater..

[47] Gevaudan J.P., Wallat J.D., Lama B., Srubar W.V. (2020). PVA- and PEG-assisted sol-gel synthesis of aluminosilicate precursors for N-A-S-H geopolymer cements. J. Am. Ceram. Soc..

[48] Ricciotti L., Occhicone A., Manzi S., Saccani A., Ferone C., Tarallo O., Roviello G. (2022). Sustainable Materials Based on Geopolymer–Polyvinyl Acetate Composites for Art and Design Applications. Polymers.

[49] Ricciotti L., Molino A.J., Roviello V., Chianese E., Cennamo P., Roviello G. (2017). Geopolymer Composites for Potential Applications in Cultural Heritage. Environments.

[50] GOST 30744–2020 CEMENTS Methods of Testing with Using Polyfraction Standard Sand. https://docs.cntd.ru/document/1200011363.

[51] GOST 12730.3–2020 Concretes Method of Determination of Water Absorption. https://docs.cntd.ru/document/1200177301.

[52] GOST R 58277–2018 Dry Building Mixes Based on Cement Binder Test Methods. https://docs.cntd.ru/document/1200162143.

[53] Huseien G.F., Sam A.R.M., Shah K.W., Mirza J., Tahir M.M. (2019). Evaluation of alkali-activated mortars containing high volume waste ceramic powder and fly ash replacing GBFS. Constr. Build. Mater..

[54] Abadel A.A., Alghamdi H. (2023). Effect of high volume tile ceramic wastes on resistance of geopolymer mortars to abrasion and freezing-thawing cycles: Experimental and deep learning modelling. Ceram. Int..

